# Determination of Interactive States of Immune Checkpoint Regulators in Lung Metastases after Radiofrequency Ablation

**DOI:** 10.3390/cancers14235738

**Published:** 2022-11-22

**Authors:** James Miles, Isabelle Soubeyran, Florence Marliot, Nicolas Pangon, Antoine Italiano, Carine Bellera, Stephen G. Ward, Franck Pagès, Jean Palussière, Banafshé Larijani

**Affiliations:** 1 Cell Biophysics Laboratory, Research Centre for Experimental Marine Biology and Biotechnology (PiE) & Instituto Biofisika (UPV/EHU, CSIC), University of the Basque Country, 48940 Leioa, Spain; 2 Early Phase Trials and Sarcoma, Institut Bergonié, Cours de l’Argonne, 33076 Bordeaux, France; 3 HAWK Biosystems, Astondo Bidea, Scientific and Technology Park of Bizkaia, 48160 Derio, Spain; 4 Molecular Pathology Unit, Department of Pathology, Institut Bergonié, Cours de l’Argonne, 33076 Bordeaux, France; 5 Immunomonitoring Platform, Laboratory of Immunology, Assistance Publique-Hôpitaux de Paris (AP-HP), Hôpital Européen Georges Pompidou, INSERM, Laboratory of Integrative Cancer Immunology, Université Paris Cité, 75006 Paris, France; 6 Interventional Radiology, Institut Bergonié, Cours de l’Argonne, 33076 Bordeaux, France; 7 Department of Clinical and Epidemiological Research, Institut Bergonié, 33076 Bordeaux, France; 8 Centre for Therapeutic Innovation, Leukocyte Biology Laboratory, Life Sciences Department, University of Bath, Claverton Down, Bath BA2 7AY, UK; 9 Centre for Therapeutic Innovation, Cell Biophysics Laboratory, Life Sciences Department, Department of Physics, University of Bath, Claverton Down, Bath BA2 7AY, UK

**Keywords:** immune checkpoints, PD-1/PD-L1, CTLA-4/CD80, radiofrequency ablation, abscopal effect, FRET/FLIM, immune surveyance

## Abstract

**Simple Summary:**

Radiofrequency ablation (RFA), when used for the treatment of pulmonary metastases, results in the generation of tumor-specific neo-antigens that may prime an immune response of the remaining metastases. Under these circumstances, the immune system may become responsible for the widespread attack of the remaining neoplasia. Immune checkpoint interactions are thought to limit this response. A platform that enables the precise measurement of immune checkpoint interactions and stratifies patients for immunotherapies, alongside radiofrequency ablation, may be a potent method for enhancing response rates in patients with metastases. To determine the immune mechanism triggered by RFA, leading to this abscopal effect, we have exploited our imaging platform that quantifies the spatio-functional states of biomarkers rather than their expression level. This platform has been tested on patients treated by RFA on lung metastases from colorectal cancer. For the first time, we have demonstrated how this platform could be used for determining the tumor and immune cell interactions in RFA patients.

**Abstract:**

Background: Cases of the spontaneous regression of multiple pulmonary metastases, after radiofrequency ablation (RFA), of a single lung metastasis, have been documented to be mediated by the immune system. The interaction of immune checkpoints, e.g., PD-1/PD-L1 and CTLA-4/CD80, may explain this phenomenon. The purpose of this study is to identify and quantify immune mechanisms triggered by RFA of pulmonary metastases originating from colorectal cancer. Methods: We used two-site time-resolved Förster resonance energy transfer as determined by frequency-domain FLIM (iFRET) for the quantification of receptor–ligand interactions. iFRET provides a method by which immune checkpoint interaction states can be quantified in a spatiotemporal manner. The same patient sections were used for assessment of ligand–receptor interaction and intratumoral T-cell labeling. Conclusion: The checkpoint interaction states quantified by iFRET did not correlate with ligand expression. We show that immune checkpoint ligand expression as a predictive biomarker may be unsuitable as it does not confirm checkpoint interactions. In pre-RFA-treated metastases, there was a significant and negative correlation between PD-1/PD-L1 interaction state and intratumoral CD3+ and CD8+ density. The negative correlation of CD8+ and interactive states of PD-1/PD-L1 can be used to assess the state of immune suppression in RFA-treated patients.

## 1. Introduction

For the treatment of unresectable primary and metastatic lung cancer, the use of radiofrequency ablation (RFA) as a minimally invasive technique was first performed 22 years ago in a human lung and, since then, its application has been more readily utilized [1,2]. In rare instances, it has been documented that the treatment of one or more tumors by RFA resulted in a reduction in tumor size in untreated tumors [3]. This phenomenon was identified over 50 years ago. The observation consisted of distant metastatic lesions regressing after radiation therapy of a primary tumor. It was postulated that the effect was due to the release of tumor-specific iso(self)-antigens creating an immune response leading to an abscopal effect. Such an effect is mediated by the development of a systematic anti-tumor immune response caused by radiation therapy [4,5]. This results in the release of intratumoral antigens adjacent to APCs and T-cells [6]. Such a phenomenon is quite similar to the immune response resulting from radiofrequency ablation. The specific or selective expression of antigens by cancer cells creates an environment for an endogenous cell-mediated serologic immune attack. Under these circumstances, immunotherapy interventions may be useful.

Nonetheless, due to the downregulation of immune activity via the checkpoint regulators, the abscopal effect remains incomplete. Thus, a combined treatment, with RFA and immune checkpoint blockade, may enhance the abscopal effect [7]. Given the uncertainty of these molecular mechanisms, there is an unmet need to identify and quantify immune mechanisms, such as the interactive states of the immune checkpoint regulators, triggered by RFA of pulmonary metastases originating from colorectal cancer.

To determine the immune mechanism triggered by RFA, we exploited an imaging platform that quantifies the functional states of biomarkers rather than their expression level. Two-site time-resolved immune-FRET (iFRET) determines the direct intercellular interactive states of immune checkpoints at distances of 1–10 nm [8,9]. This yields a measurement of the functionality of a biomarker (PD-1/PD-L1 or CTLA-4/CD80 in this instance) rather than its expression. We sought to apply iFRET to directly quantify PD-1/PD-L1 and CTLA-4/CD80 interactions in a cohort of patients in both their pre- and post-RFA-treated lung metastases, and iFRET results were correlated with the quantification of tumor-infiltrating lymphocytes.

This is the first time that CTLA-4/CD80 interactions have been quantified in situ in patient samples at a 1–10 nm spatial resolution. iFRET quantified a significant amount of inter- and intratumoral heterogeneity across both checkpoint pathways. We show that PD-1/PD-L1 interaction negatively correlated with intratumoral CD3 and CD8 density.

This is crucial, as it is the functionality of a biomarker that drives disease progression, as opposed to biomarker expression. Previously, iFRET was able to functionally quantify PD-1/PD-L1 interactions and we found this interaction state to be predictive of patient outcome in malignant melanoma and metastatic NSCLC. In this latter study, PD-L1 expression was not predictive of patient outcome [8]. This study amongst others utilizing two-site time-resolved amplified FRET highlights the unmet need for the quantification of biomarker functionality as opposed to biomarker expression.

## 2. Materials and Methods

### 2.1. Patient Population RFA

This study (NCT 03960021) was approved by the Institutional Review Board of the Institut Bergonié (Bordeaux, France) and its ethics committee. The study was funded by the “groupe interregional de recherche Clinique et d’innovation (GIRCI)” and by Boston Scientific. Out of 20 patients, only 15 were useable. The 15 patients consisted of nine males and six females with a median age of 73.5 years (32–89) with bilateral metastasis originating from colorectal cancer. None or slowly progressive, with or without chemotherapy, were included. Of note, 17 were evaluated for the primary endpoint; three patients were not evaluated for the primary endpoint because they were noncontributing samples. The third patient had a quick progression of the metastatic disease before the second radiofrequency ablation. Furthermore, the influence of chemotherapy was not tested as patients were not naïve; they already had different chemotherapy regimens before RFA treatment. Most of the patients in this series were treated for a chronic metastatic disease. RFA was proposed to provide a chemotherapy “holiday”. The exposure to persistent tumoral antigenic stimulation may induce a dysfunctional state called “exhaustion” where cytotoxic CD8+T cells are attenuated and fail to control tumor progression [10].

All patients were microsatellite stable (MSS). Due to bilateral pulmonary metastatic disease, each patient was treated twice: two RFA interventions (one from each lung) with 4–6 weeks between the two RFA procedures. A biopsy of one metastasis was performed at each RFA session before heating the metastases with RFA. Four sections were provided per patient prior to the radiofrequency ablation procedure being carried out in the first lung (lung one) and four weeks later on the contralateral lung (lung 2); another four sections were provided per patient from lung two (Supplementary Figure S1).

Biopsied samples were sent to the Bio-Pathology Department at the Institut Bergonié for a comparative measurement before and after RFA. Samples were FFPE in tissue blocks prior to being sequentially sectioned and mounted on microscope slides.

Thermal ablation was achieved with an impedance-based feedback generator delivering an output frequency of 460 kHz (RF3000; Boston Scientific, Natick, MA, USA), with LeVeen CoAccess multitined expandable 14-gauge electrodes (Boston Scientific), and with a 15 cm long, 14-gauge insulated diamond-tip guiding needle. Prone, supine, or lateral positions were used to obtain the shortest access route to the tumor and also to give access to all metastases. Biopsies were performed through the guiding needle with a 20 cm long 18-gauge biopsy gun (Bard BD Franklin Lakes, NJ, USA) before introducing the electrode.

### 2.2. Antibodies and Reagents

Monoclonal antibodies mouse anti-PD-1, rabbit anti-PD-L1, and mouse anti-CTLA-4 were purchased from Abcam (catalogue numbers ab52587, ab205921, and ab19792, respectively). Polyclonal rabbit anti-CD80 was purchased from MyBioSource (catalogue number MBS2522916). AffiniPure F(ab’)_2_ fragment donkey anti-mouse IgG (H+L) and peroxidase AffiniPure F(ab’)_2_ fragment donkey anti-rabbit IgG (H+L) were purchased from Jackson Immuno Research (catalogue numbers 715-006-150 and 711-036-152, respectively). Pierce endogenous peroxidase suppressor, TSA SuperBoost kit, and a Prolong Glass antifade mount were purchased from Thermofisher Scientific (catalogue numbers 35000, B40925 and P36980 respectively). Sodium borohydride, ATTO488 NHS ester, bovine serum albumin, and rhodamine B were purchased from Sigma Aldrich (catalogue numbers 41698-1MG-F, A2153-100G, and 234141-10G, respectively).

### 2.3. Time-Resolved Immune FRET (iFRET) Assay Determined by Frequency-Domain FLIM

iFRET is a quantitative molecular imaging platform that relies on a two-site labeling assay to directly quantify immune checkpoint interactions at a nanoscopic resolution (Supplementary Figure S2). Two primary antibodies were used to detect the receptor (PD-1 or CTLA-4) and ligand (PD-L1 or CD80), respectively. These antibodies were labeled with species-specific F(ab’)_2_ fragments that were conjugated to ATTO488 (donor chromophore, used to label the receptor primary antibody) or HRP (used to label the ligand primary antibody). Tyramide signal amplification was used to conjugate the acceptor chromophore (Alexa594) to the HRP labeling the ligand. This reaction is outlined in Supplementary Figure S3.

One sample per patient condition was labeled with only the donor primary and secondary antibodies. The subsequent slide per patient condition was labeled with both the donor and acceptor primary and secondary antibodies. This was repeated for both checkpoints analyzed. The first slide (donor only) was excited by a 473 nm laser, and the lifetime of the donor alone was recorded. The second slide was excited by the 473 nm laser and the lifetime of the donor in the presence of the acceptor was recorded. The reduction in the donor lifetime (caused by resonance energy transfer) that was due to the presence of the acceptor reports in distances of 1–10 nm and therefore acts as a “chemical ruler” that enables the quantification of receptor–ligand interactions.

Our custom-made semi-automated high-throughput multiple-frequency FLIM microscope (Nikon Ti2) created a mapping file, mapping regions of interest on a slide. This mapping file was automatically acquired, and phase lifetimes, donor and acceptor intensities, and lifetime images were calculated. These were automatically exported to an Excel spreadsheet. A decrease in donor lifetime in the presence of the acceptor chromophore is indicative of resonance energy transfer. FRET efficiency (Ef) values were calculated using the following equation, where τ_D_ and τ_DA_ are the lifetimes of the donor alone and in the presence of the acceptor, respectively:

Ef = (1 − τ_DA_/τ_D_) × 100

The Förster radius (R_0_) of ATTO488 and Alexa594 is 5.83 nm. This is the minimum permitted distance between this chromophore pair where energy transfer is maximal. At this distance, FRET efficiency is 50%, the highest permitted value. Of note, only high signal-to-noise-acceptor fluorescence intensities were used to validate the decrease in donor lifetime.

### 2.4. iFRET Labelling Assay for PD-1/PD-L1 and CTLA-4/CD80 in RFA-Treated Lung Metastases

Four FFPE tissue sections per patient, both pre- and post-RFA treatment, were mounted on separate glass microscope slides. This allowed for donor-only and donor-acceptor labeling to be carried out for all patient samples, both pre- and post-RFA treatment. Both checkpoints were analyzed per patient. Samples underwent dewaxing and antigen retrieval using Envision Flex retrieval solution (pH 9) in a Dako PT-Link. Here, slides are heated to 95 °C for 20 min. The remaining paraffin was removed by washing the slides three times with PBS in a Coplin jar with gentle agitation. A hydrophobic border was drawn around each piece of tissue using a PAP pen. Fresh sodium borohydride solution (1 mg/mL) was added to each slide and incubated at room temperature for 10 min to quench tissue autofluorescence. Two drops of Pierce endogenous peroxidase suppressor were added to each sample and the slides incubated for 30 min at room temperature in a humidified tray. Samples were washed three times with PBS prior to incubation with 1% BSA (10 mg/mL) for one hour at room temperature. Donor-only samples were incubated with αPD-1 (1:100) or αCTLA-4 (1:100). Donor-acceptor samples were incubated with αPD-1 (1:100), or αCTLA-4 (1:100) and αPD-L1 (1:500), or αCD80 (1:500). Samples were incubated overnight at 4 °C.

Samples were removed from the refrigerator and washed three times with 0.02% PBST (PBS with Tween20). Secondary F(ab’)_2_ fragments were added to the samples. F(ab’)_2_-ATTO488 (1:50) was added to the donor-only slides. F(ab’)_2_-ATTO488 (1:50) and F(ab’)_2_-HRP (1:200) were added to the donor-acceptor slides.

Samples were incubated in the dark at room temperature in humidified trays for two hours. Slides were washed three times with 0.02% PBST. Donor-only slides were washed once more with PBS and mounted with 1–2 drops of Prolong Glass antifade mount. The edges of each slide were sealed with clear nail varnish. Donor-acceptor slides underwent tyramide signal amplification (TSA). The purpose of tyramide signal amplification was to amplify the acceptor labeling, thus increase the signal-to-noise ratio and enhance the resonance energy transfer. Briefly, the reaction involves adding tyramide-Alexa594 to the samples in the presence of hydrogen peroxide. Tyramide-Alexa594 is a phenol ring with an Alexa594 chromophore at the para position to the hydroxyl group. The hydrogen peroxide, in the presence of HRP (labeling the acceptor antibody), causes the formation of a free radical on the phenol ring of the tyramide, which subsequently reacts and covalently binds to nearby tyrosine residues in the sample on the F(ab’)_2_ fragment. This reaction was carried out for 20 min at room temperature in the dark in a humidified tray. Slides were washed three times with 0.02% PBST in Coplin jars before a final PBS wash. Slides were mounted with Prolong Glass and sealed as described above.

### 2.5. Intratumoral CD3 and CD8 Labelling

Briefly, two formalin-fixed paraffin-embedded tissue sections of 4 mm were processed for immunohistochemistry (IHC) with antibodies against CD3+ (clone 2GV6, Ventana) and CD8+ (clone C8/144B, Dako). The pathologist reviewed all biopsies for delimitation of the tumoral component. Stained cells of the tumor were quantified using a previously validated module of the Developer XD image analysis software (Definiens Inc, Carlsbad, CA 92008, United States).

### 2.6. Statistical Analysis

The normality of the data was tested using the Sharipo–Wilk test and the data confirmed to be nonparametric. Box and whisker plots were drawn using GraphPad Prism 9. Boxes represent the 25–75% range of the data with the whiskers representing the 1–99% range. Regression curves plotting intratumoral lymphocyte density versus checkpoint interaction were also drawn using GraphPad Prism 9 and the r values were calculated. As the datasets were determined to be nonparametric, Spearman’s regression values (r_s_) were calculated to determine correlations between checkpoint interaction and CD3 or CD8 infiltration.

## 3. Results

### 3.1. Patient Population

Twenty patients were included across one single center from March 2019 to February 2021. The colorectal origin of the metastases and tumor characteristics were confirmed for all patients by histopathologic analysis. Twenty patients met the inclusion criteria, 20 were eligible, and 17 evaluated for the primary endpoint. Median patient age was 73.5 years and 60.9% were female. When labeled with two-site assay reagents, only 15 out of 20 patient samples suited the criteria for FRET analysis.

### 3.2. Validation of CTLA-4/CD80 Quantification in Tissue

Prior to utilizing iFRET to quantify CTLA-4/CD80 interaction states in RFA-treated lung metastases, we sought to validate the use of this labeling antibody pair for use in tissue with iFRET. The CTLA-4/CD80-labeling antibodies used were validated with our iFRET assay in a cell-coculture assay [8]. However, these antibodies had not been validated for use in tissue samples. Prior to their use to quantify CTLA-4/CD80 interactions in RFA-treated lung tissue, the concentrations used in the cell assay were validated in tissue. A commercial TMA was purchased that contained duplicated cases of common lung cancer types. In total, the TMA contained 150 cores. Two slides were used, one donor-only and one donor-acceptor. The antibody dilutions used were the same as those used in the cell co-culture assay of Sánchez-Magraner et al., 2020 (1:100 for both antibodies).

Supplementary Figure S4 highlights the range of FRET efficiencies determined when using the CTLA-4/CD80 antibody pair in these TMAs. A range of FRET efficiencies were determined across the tumor types, which were classified as either non-small-cell lung carcinoma (NSCLC) or small-cell lung carcinoma (SCLC). The median FRET efficiency values obtained were 30.62% and 29.19% for the NSCLC and SCLC, respectively. The highest FRET efficiency observed in the NSCLC and SCLC groups were 42.79% and 36.23%, respectively. In both box and whisker plots, a population of high FRET efficiencies were detected; these are values in the upper quartile of the plot. No significant differences in CTLA-4/CD80 interaction states were observed between the two groups.

Whilst our automated FLIM platform acquired 150 cores per slide, only those with high coincidence between the donor and donor-acceptor, and an appropriate phase lifetime standard deviation, were analyzed further and plotted in the box and whisker plot. The two-site coincidence nature of our assay requires a suitable spatial coincidence between the donor and donor-acceptor slide to correctly analyze the spatiotemporal change in donor lifetime in the presence of the acceptor. The signal observed, combined with the range of positive FRET efficiencies, indicated that the chosen antibody dilutions, previously used in cells by Sánchez-Magraner et al., 2020, were suitable for use in tissue. Therefore, subsequent experiments using pre- and post-RFA-treated lung biopsies were analyzed with these antibody dilutions.

### 3.3. iFRET Quantifies Both PD-1/PD-L1 and CTLA-4/CD80 Interaction States in Pre-RFA Treated Samples

We analyzed data as either pre-RFA treatment or post-RFA treatment and compared PD-1/PD-L1 and CTLA-4/CD80 interaction states within these groups.

**Figure 1:**
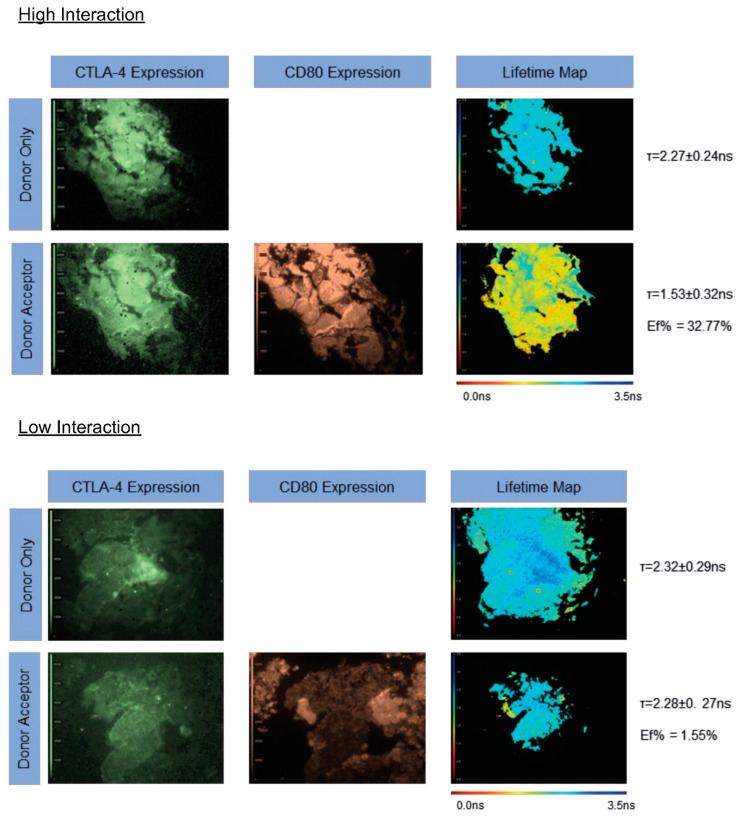
iFRET quantifies high and low PD-1/PD-L1 interaction states in lung metastases, detecting inter- and intratumoral heterogeneity. Representative FLIM images demonstrating high and low CTLA-4/CD80 interaction states. Top panel: greyscale images indicate CTLA-4 or CD80 expression in donor-only or donor-acceptor tissue slices for one patient. No difference is observed between CTLA-4 expression between the donor-only and donor-acceptor slides. The lifetime map indicates the mean lifetime per pixel of an image. The donor only lifetime (2.27 ± 0.24 ns) is represented by blue/green in the pseudo-color scale. In the donor-acceptor slide, the lifetime of the donor is reduced to 1.53 ± 0.32 ns, yielding a FRET efficiency of 32.77%. This is indicative of a high CTLA-4/CD80 interaction state. Bottom panel: here, a good expression of CTLA-4 and CD80 are observed and no significant differences in expression profiles are seen between the top and bottom panels. The donor-only lifetime here is 2.32 ± 0.29 ns, which is reduced to 2.28 ± 0.27 ns in the presence of the acceptor, giving a FRET efficiency of 1.55%. This indicates that CTLA-4/CD80 are undergoing little to no interaction in this sample, despite the presence of both receptor and ligand. Critically, in both examples, high tissue level coincidence is observed between the donor-only and donor-acceptor slices, meaning that donor lifetime changes are due to the presence of the acceptor chromophore and are not reporting on intratumoral heterogeneity.

**Figure 2:**
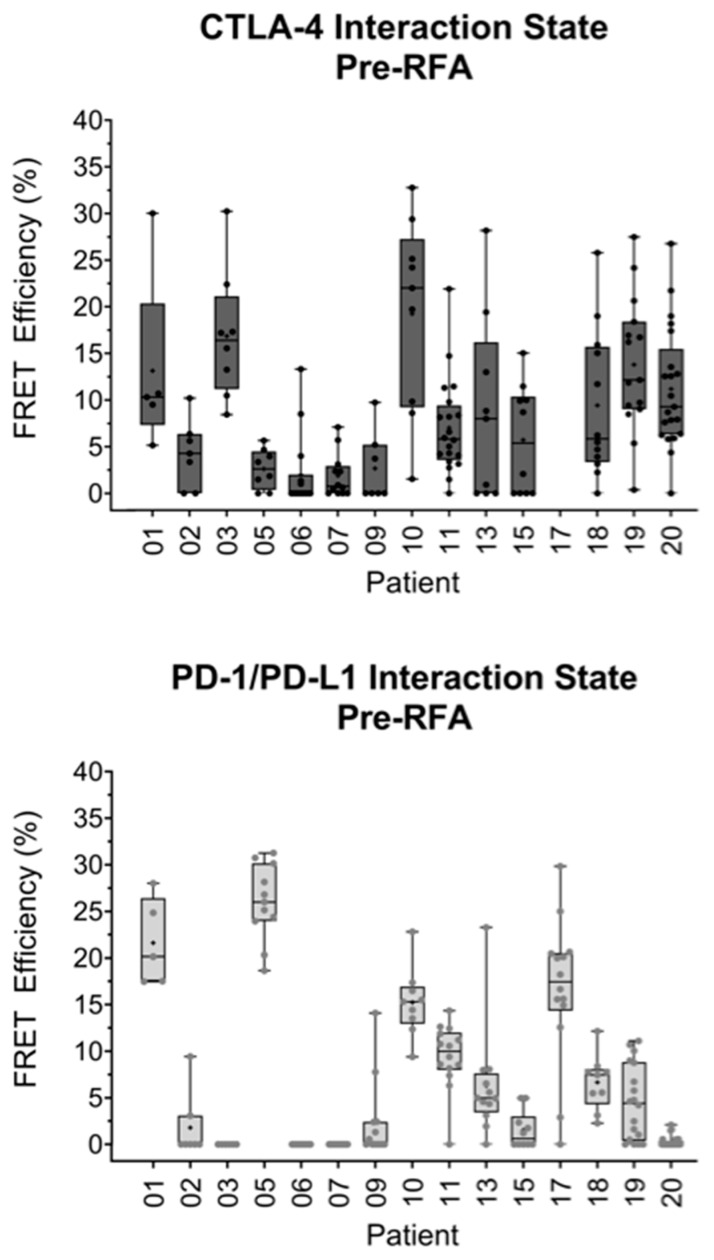
iFRET detects intra- and intertumoral heterogeneity in both CTLA-4/CD80 and PD-1/PD-L1 interactions in metastases from lung one (pre-RFA). Box and whisker plots show the statistical distribution of all interaction states recorded for CTLA-4/CD80 (top panel) and PD-1/PD-L1 (bottom panel) in metastases taken from lung one, before RFA was carried out. Each point represents one region of interest for an analyzed patient sample. Note, no data were analyzed for CTLA-4/CD80 interactions in patient 17 due to poor a poor signal-to-noise ratio. Interaction states are generally higher in CTLA-4/CD80 than PD-1/PD-L1 with two patients having no PD-1/PD-L1 interaction across the sample. Some patients have differential interaction profiles for the two pathways. Patient 05 has a low CTLA-4/CD80 interaction and high PD-1/PD-L1 interaction. A contrasting trend is seen in patient 20, who has a significantly higher CTLA-4/CD80 interaction state than PD-1/PD-L1. Some patients, such as patient 13, showed no significant differences between CTLA-4/CD80 and PD-1/PD-L1 interaction states.

We first determined the interaction state of CTLA-4/CD80 in 15 lung metastases from lung number one before the RFA procedure was carried out. One sample (17SD RF1) showed a consistently poor signal-to-noise ratio of less than four (Figure 1), so was not further analyzed for CTLA-4/CD80 interaction or CD80 expression (Figure 2).

Figure 1 shows representative FLIM images for patient 10 pre-RFA (lung 1). The top panel indicates a region of interest with a high CTLA-4/CD80 interaction state. The pseudo-color fluorescent intensity images represent CTLA-4 and CD80 expression and the lifetime maps indicate the average donor lifetime of each pixel. The pseudo-color lifetime scale runs from 0.00 ns (red) to 3.50 ns (blue). In this example, the lifetime of the donor alone is 2.27 ± 0.24 ns, which was reduced to 1.53 ± 0.32 ns in the presence of the acceptor. This yields a FRET efficiency of 32.77%. In the bottom panel, the donor-only lifetime was 2.32 ± 0.29 ns, reduced to 2.28 ± 0.27 ns in the presence of the acceptor. This yields a FRET efficiency of 1.55% and indicates an insignificant interaction state (>10 nm). In both examples, good coincidence was seen between the donor-only and donor-acceptor slides. In both instances, no difference was observed in either CTLA-4 or CD80 expression, whereas the interaction state is significantly different in both cases (Figure 1).

All CTLA-4/CD80 interactions quantified from metastases in lung one, prior to RFA treatment, were plotted on a box and whisker plot (Figure 2, top panel). The highest interaction state observed was 32.77% in patient 10. The lowest median FRET efficiency of 0.00% was observed in patients 06 and 09. The highest median interaction state of 22.01% was detected in patient 10.

**Figure 3:**
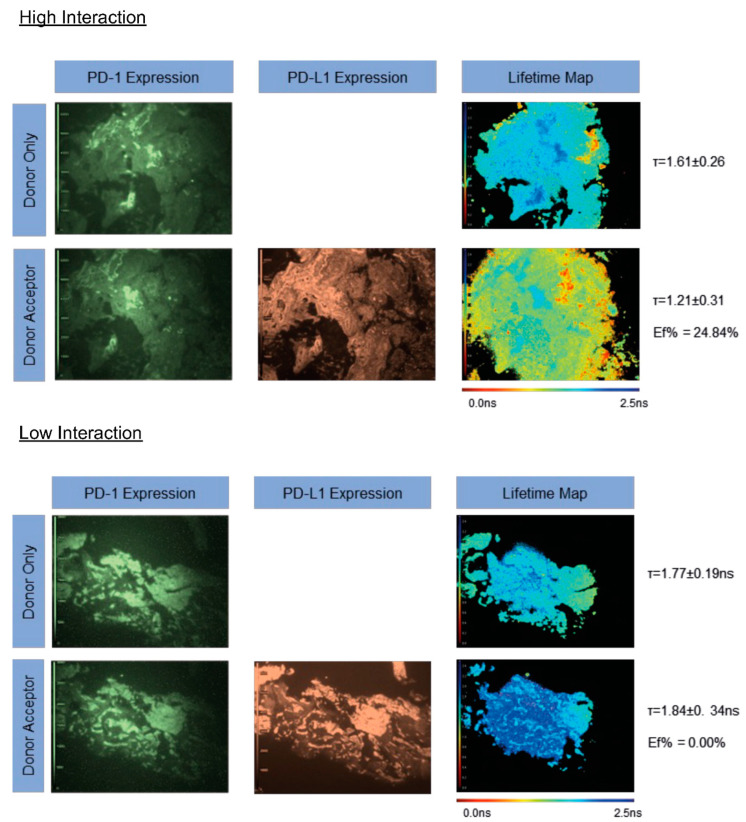
iFRET quantifies high and low PD-1/PD-L1 interaction states in lung metastases, detecting inter- and intratumoral heterogeneity. Representative FLIM images demonstrating high and low PD-1/PD-L1interaction states. Top panel: greyscale images indicate PD-1 or PD-L1 expression in donor-only or donor-acceptor tissue slices for one patient. No difference is observed between PD-1 expression between the donor-only and donor-acceptor slides. The lifetime map indicates the mean lifetime per pixel of an image. The donor-only lifetime (1.61 ns ± 0.26 ns) is represented by blue/green in the pseudo-color scale. In the donor-acceptor slide, the lifetime of the donor is reduced to 1.21 ± 0.31 ns, yielding a FRET efficiency of 24.84%. This is indicative of a high PD-1/PD-L1 interaction state. Bottom panel: here, a good expression of PD-1 and PD-L1 are observed and no significant differences in expression profiles are seen between the top and bottom panels. However, the donor-only lifetime here is 1.77 ± 0.19 ns, which fails to reduce in the presence of the acceptor, giving a FRET efficiency of 0.00%. This indicates that PD-1/PD-L1 are not interactive in this sample, despite the presence of both receptor and ligand. Critically, in both examples, high tissue level coincidence is observed between the donor-only and donor-acceptor slices, meaning that donor lifetime changes are due to the presence of the acceptor chromophore and are not reporting on intratumoral heterogeneity.

PD-1/PD-L1 interaction was determined in the same 15 lung metastases from lung one before the RFA procedure was performed. Figure 3 shows representative FLIM images for high and low FRET efficiencies within patient 01. The pseudo-color fluorescent intensity images represent PD-1 or PD-L1 expression and the lifetime map shows the donor lifetime, per pixel, of the sample with a pseudo-color scale. In the pseudo-color scale, blue represents a high lifetime (2.5 ns) and red represents a low lifetime (0.0 ns). In a pre-RFA-treatment sample of patient 01, a significant donor (PD-1) intensity was observed on both the donor and donor-acceptor slides, with a strong acceptor (PD-L1) expression also on the acceptor slide. The donor-only lifetime was 1.61 ± 0.26 ns, which was reduced to 1.21 ± 0.31 ns in the presence of the acceptor. This resulted in a FRET efficiency of 24.84%. In a post-RFA-treatment sample of patient 01, no PD-1/PD-L1 interaction was observed. Again, a strong PD-1 and PD-L1 expression profile was observed across the sample; however, in this instance the donor-only lifetime (1.77 ± 0.19 ns) failed to decrease in the presence of the acceptor, yielding a FRET efficiency of 0.00% (Figure 3). In both FLIM images, tissue coincidence was observed between the donor and donor-acceptor slides.

All PD-1/PD-L1 interaction states were plotted in a box and whisker plot (Figure 2, bottom panel). The highest FRET efficiency observed, 31.25%, was in patient 05. The highest median FRET efficiency observed, also in patient 05, was 26.00%. A large degree of intra- and intertumoral heterogeneity was observed across the samples. When comparing the CTLA-4/CD80 and PD-1/PD-L1 interactions, the CTLA-4/CD80 and PD-1/PD-L1 interactions were similar, although more patients had a net zero FRET efficiency in the PD1/PD-L1 group (Figure 2). When comparing patients, some changes between the two pathways were detected. Patient 05 has a lower CTLA-4/CD80 interaction state (median FRET efficiency 2.62%) than PD-1/PD-L1 interaction state (median FRET efficiency 26.00%). A contrasting trend is observed with patient 20, with CTLA-4/CD80 interactions being higher (median FRET efficiency 9.28%) than PD-1/PD-L1 interaction (median FRET efficiency 0.00%). Some patients, such as patient 13, showed no significant difference in checkpoint interaction state between CTLA-4/CD80 (median FRET efficiency 8.00%) and PD-1/PD-L1 (median FRET efficiency 4.98%).

### 3.4. iFRET Quantifies Both PD-1/PD-L1 and CTLA-4/CD80 Interaction States in Post-RFA-Treated Samples

Following the quantification of the CTLA-4/CD80 and PD-1/PD-L1 interaction states in the metastases of lung one, pre-RFA treatment, we analyzed the immune checkpoint interaction states in biopsies from lung two, post-RFA treatment of lung one. Of the 15 patients analyzed above, 13 had biopsies taken from lung two, post-RFA treatment of lung one.

**Figure 4:**
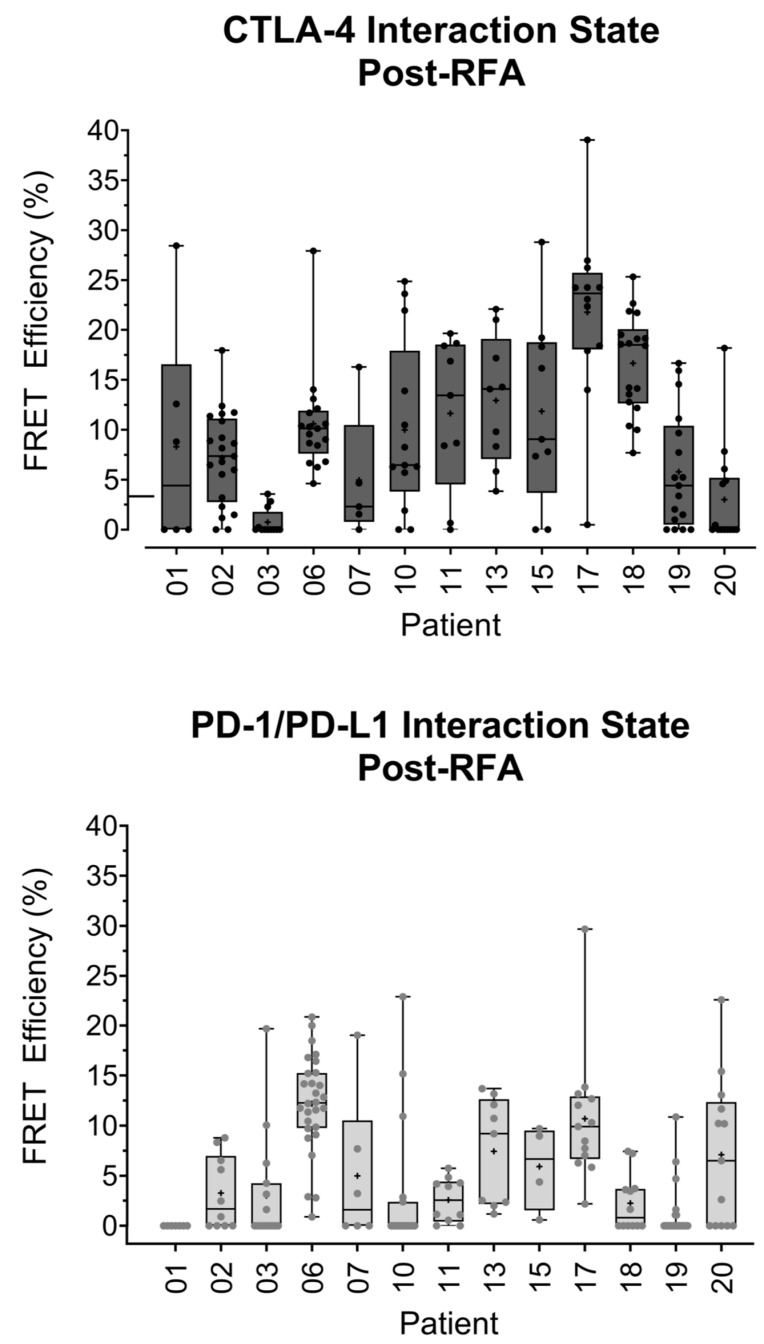
iFRET detects intra- and intertumoral heterogeneity in both CTLA-4/CD80 and PD-1/PD-L1 interactions in metastases from lung two (post-RFA). Box and whisker plots show the statistical distribution of all interaction states recorded for CTLA-4/CD80 (left-hand side) and PD-1/PD-L1 (right-hand side) in metastases taken from lung two, after RFA was carried out in lung one. Each point represents one region of interest for an analyzed patient sample. Interaction states are generally higher in CTLA-4/CD80 than PD-1/PD-L1. Some patients have differential interaction profiles for the two pathways. Patient 03 has a low CTLA-4/CD80 interaction state and regions of high PD-1/PD-L1 interaction. A contrasting trend is seen in patient 01, who has a significantly higher CTLA-4/CD80 interaction state than PD-1/PD-L1. Some patients, such as patient 06, showed no significant differences between CTLA-4/CD80 and PD-1/PD-L1 interaction states.

Figure 4, top panel, is a box and whisker plot analyzing the CTLA-4/CD80 and PD-1/PD-L1 interaction states observed in the 13 patients. Here, PD-1/PD-L1 interactions are lower than CTLA-4/CD80 interactions with one PD-1/PD-L1 patient having a net zero FRET efficiency. The highest CTLA-4/CD80 interaction state observed was 39.05% in patient 17. The highest PD-1/PD-L1 interaction observed, also in patient 17, was 29.66%. The highest median FRET efficiency observed in the CTLA-4/CD80 group was 23.66% in patient 17. The highest median FRET efficiency observed in the PD-1/PD-L1 group was 12.28% in patient 06. Direct patient comparisons revealed that patient 01 had a higher CTLA-4/CD80 interaction state (median FRET efficiency 4.41%) than PD-1/PD-L1 interaction state (median FRET efficiency 0.00%) (Figure 4, bottom panel). Patient 03 showed the opposite trend with a lower CTLA-4/CD80 interaction state (median FRET efficiency 0.00%) than PD-1/PD-L1 interaction state (median FRET efficiency 0.00%). Whilst the median values for patient 03 were both 0.00%, the box and whisker plots show three regions with higher PD-1/PD-L1 interactions than CTLA-4/CD80 interactions. Patient 06 showed no significant difference between checkpoint interaction states with a similar CTLA-4/CD80 (median FRET efficiency 10.13%) and PD-1/PD-L1 (median FRET efficiency 12.23%) interaction states.

### 3.5. Immune Checkpoint Ligand Expression Does Not Correlate with Immune Checkpoint Engagement

Current attempts to utilize immune checkpoints as predictive biomarkers have relied on ligand expression as assessed by immunohistochemistry. Sánchez-Magraner et al., 2020, identified that PD-L1 expression was not predictive of patient outcome, whereas the PD-1/PD-L1 interaction state was predictive in malignant melanoma and NSCLC [8]. Whilst this is a prospective study (with no patient outcomes available), we aimed to identify if there were a correlation between ligand expression and checkpoint interaction. The first correlations assessed checkpoint interaction versus ligand expression in biopsies from lung one, prior to the RFA treatment of lung one.

**Figure 5:**
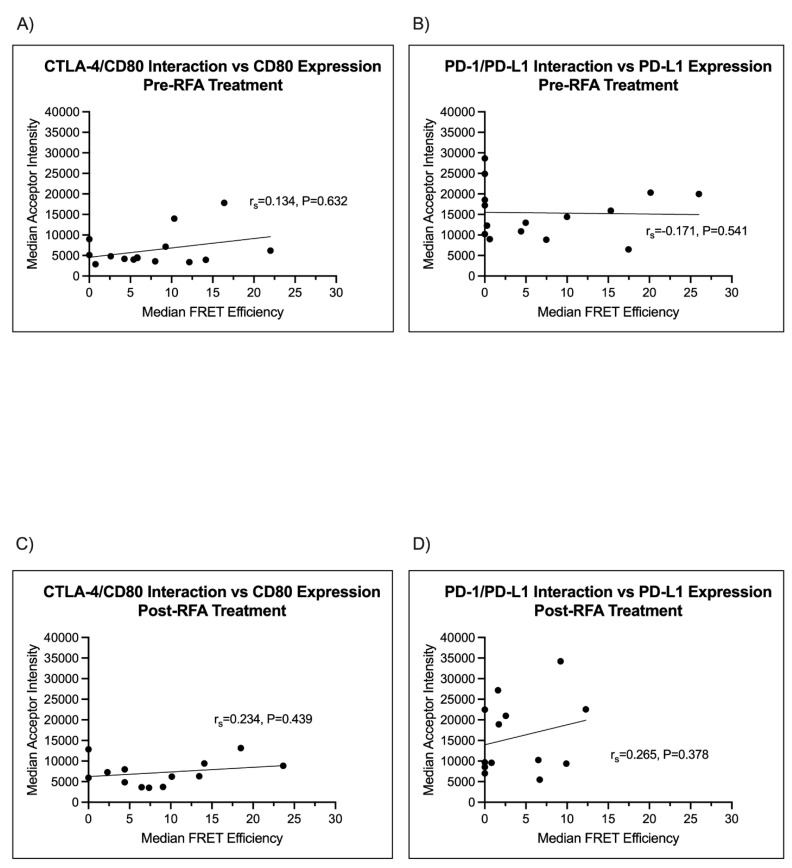
Checkpoint interaction does not correlate with ligand expression in pre- or post-RFA-treated lung metastases. The expression of each checkpoint ligand (CD80 or PD-L1) does not correlate with checkpoint interaction. (**A**) In lung metastases from lung 1, prior to RFA treatment, median CTLA-4/CD80 interaction states did not correlate with CD80 expression (r_s_ = −0.134, *p* = 0.632). (**B**) From the same lung, pre-RFA, PD-1/PD-L1 interaction states did not correlate with PD-L1 expression (r_s_ = −0.171, *p* = 0.541). (**C**) In patient samples taken from lung 2, post-RFA treatment of lung 1, checkpoint ligand expression failed to correlate with median CTLA-4/CD80 interaction (r_s_ = 0.234, *p* = 0.439). (**D**) PD-1/PD-L1 interaction states failed to correlate with PD-L1 expression (r_s_ = −0.265, *p* = 0.378, respectively).

The scatter plots shown in Figure 5A correlated CTLA-4/CD80 interaction and CD80 expression. The x axis indicates the checkpoint interaction (FRET efficiency) and the y axis is the acceptor chromophore intensity (arbitrary units) as a surrogate of CD80 expression. An r_s_ value of 0.134, *p* = 0.632, indicated no significant correlation between CD80 expression and the CTLA-4/CD80 interaction state. Figure 5B illustrates the correlation between the PD-1/PD-L1 interaction state, as measured by FRET efficiency, and PD-L1 expression, as measured by acceptor chromophore intensity. An r_s_ value of −0.171, *p* = 0.541, again indicates a lack of correlation between the PD-1/PD-L1 interaction state and PD-L1 expression.

Next, we correlated checkpoint interaction and ligand expression in biopsies from lung two, after the RFA treatment of lung one had been performed. Figure 5C correlated the CTLA-4/CD80 interaction state with CD80 expression. The correlation was r_s_ = 0.234, *p* = 0.439, indicating no correlation between CTLA-4/CD80 interaction and CD80 expression. Figure 5D demonstrates that there is no correlation between PD-1/PD-L1 and PD-L1 expression (r_s_ = 0.265, *p* = 0.378) in the second lung, after treatment of lung one. Together, these data confirm that ligand expression does not corroborate immune checkpoint interaction, as was shown in Sánchez-Magraner et al., 2020.

### 3.6. Intratumoral CD3 and CD8 Densities Negatively Correlate with PD-1/PD-L1 Interaction

Whilst an abscopal effect was not observed clinically in this patient cohort, a change in immune-cell infiltration between RFA treatments cannot be ruled out. Therefore, we proceeded to correlate CTLA-4/CD80 and PD-1/PD-L1 interaction states with intratumoral CD3 and CD8 cell infiltration. The following n numbers were available for the immune-infiltration analyses presented below: CTLA-4/CD80 Pre-RFA (n = 13); PD-1/PD-L1 Pre-RFA (n = 14); CTLA-4/CD80 Post-RFA (n = 12); and PD-1/PD-L1 Post-RFA (n = 12).

**Figure 6:**
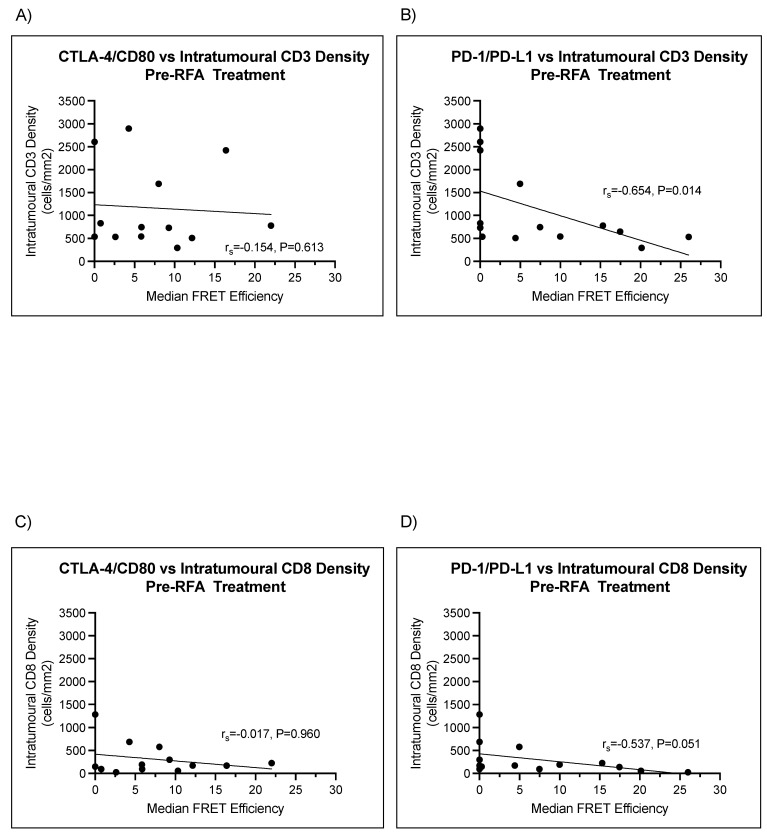
PD-1/PD-L1 interaction state negatively correlates with intratumoral CD3 and CD8 density in lung 1. **(A)** Scatter plots demonstrate a lack of correlation between intratumoral CD3 density (cells/mm^2^) and median CTLA-4/CD80 interaction (r_s_ = −0.154, *p* = 0.613) in metastases analyzed from lung one (pre-RFA). (**B**) A moderate negative correlation exists between intratumoral CD3 density and median PD-1/PD-L1 interaction state (r= −0.654, *p* = 0.014). This indicates, with significance, that higher median PD-1/PD-L1 interaction states are detected in areas with lower intratumoral CD3 density. (**C**) Scatter plots demonstrate a lack of correlation between intratumoral CD8 density (cells/mm^2^) and median CTLA-4/CD80 interaction (r_s_ = −0.017, *p* = 0.960) in metastases analyzed from lung one (pre-RFA). (**D**) A moderate negative correlation exists between intratumoral CD8 density and median PD-1/PD-L1 interaction state (r_s_ = −0–537, *p* = 0.051). Whilst not statistically significant, this is a moderate trend that may achieve significance with higher sample numbers.

We correlated intratumoral CD3 and CD8 density with median PD-1/PD-L1 and CTLA-4/CD80 interaction states in both pre- and post-RFA-treated lung metastases. No correlation was observed between median CTLA-4/CD80 interaction and intratumoral CD3 density in patients pre-RFA treatment (r_s_ = −0.154, *p* = 0.613) (Figure 6A). Median PD-1/PD-L1 interaction state moderately and negatively correlated with intratumoral CD3 density in pre-RFA-treated metastases (lung 1). Figure 6B shows a negative moderate correlation between median PD-1/PD-L1 interaction states and intratumoral CD3 density (r_s_ = −0.654, *p* = 0.014). Moreover, no significant correlations existed between lymphocyte infiltration and median CTLA-4/CD80 interaction in pre-RFA-treated samples (r_s_ = −0.017, *p* = 0.960) (Figure 6C). CD8 intratumoral density moderately and negatively correlated with intratumoral CD8 density (r_s_ = −0.537, *p* = 0.051) (Figure 6D).

No correlations were observed between median-checkpoint interaction states and intratumoral CD3 density in patients after RFA (r_s_ = −0.112, *p* = 0.733 and r_s_ = 0.155, *p* = 0.632, respectively) (Supplementary Figure S5A,B). Moreover, no correlations existed between either median PD-1/PD-L1 and median CTLA-4/CD80 interaction states and lymphocyte infiltration in post-RFA-treated samples from lung 2 (r_s_ = −0.182, *p* = 0.573 and r_s_ = −0.014, *p* = 0.973, respectively) (Supplementary Figure S5C,D).

## 4. Discussion

PD-L1 expression in colorectal cancer is relatively lower than with other cancer types such as melanoma, renal cancer, and lung cancer [11]. Nevertheless, it has been demonstrated that RFA-treated liver metastases from colorectal cancer upregulated PD-L1 expression, which is associated with an increase in T-cell infiltration in primary colorectal cancer [12]. Consequently, RFA could increase the number of potential candidates for anti-PD-L1-PD-1 therapy as RFA becomes a more utilized therapy for the treatment of lung metastases [13], potentially in combination with immune checkpoint blockade. As we have shown in Magraner et al. 2020, iFRET can be used to determine the interaction state of checkpoint regulators [8]. We therefore deployed iFRET to examine the interactive states of checkpoint regulators in RFA-treated patients. In an initial cohort of RFA-treated patients, with samples both pre- and post-RFA treatment, we utilized iFRET to quantify PD-1/PD-L1 and CTLA-4/CD80 interactions. It was observed that iFRET detects a large inter- and intratumoral heterogeneity of interaction states of both PD-1/PD-L1 and CTLA-4/CD80. Moreover, we observed that the PD-1/PD-L1 interaction state moderately and negatively correlated to intratumoral CD3 and CD8 density in the pre-RFA-treated metastases.

This work has quantified the PD-1/PD-L1 interaction states in lung metastases and directly quantified CTLA-4/CD80 engagement in the same patient samples for the first time. This has resulted in the validation of an assay that can precisely quantify differences in immune checkpoint interaction states between patients. The determination of an accurate stratification of patients can allow for the precise treatment of immune checkpoint-blocking drugs to a stratified patient subset. The results presented in Figure 2 and Figure 4 highlight the ability of this assay to determine the differences between checkpoint engagements in a single patient. The differences observed between the two pathways could be a result of the biological roles of each checkpoint. The CTLA-4/CD80 checkpoint is more prominent in early immune responses. In contrast, PD-1/PD-L1 signaling is associated with a later immune response and is linked to T-lymphocyte exhaustion that is due to chronic antigen exposure, a phenotype often seen in tumor-infiltrating lymphocytes [14]. The increased prominence of PD-1/PD-L1 signaling in the tumor microenvironment could in part explain why no correlations were observed between lymphocyte infiltration and CTLA-4/CD80 checkpoint interaction. However, whilst any one tumor may evade the host immune response by engaging one immune checkpoint, this does not mean a tumor will fully succumb to a blockade of this checkpoint. The use of a monotherapy against either PD-1/PD-L1 or CTLA-4/CD80 may apply a positive selection pressure to a tumor, resulting in the outgrowth of clones that are able to modulate other immune checkpoints. Therefore, iFRET, in combination with other spatiotemporal imaging methods [15], may be utilized to stratify patients who would respond to initial mono- or dual therapies; a continual monitoring of tumor-immune cell interactions would be required to screen for an evolution of immune evasion. Additionally, the initial and simultaneous assessment of several immune checkpoints could yield important data that may further guide patient treatment regimens.

iFRET has shown its potential value as a patient stratification tool; it also yields more information compared to the current patient stratification readout: PD-L1 expression. iFRET is capable of measuring intercellular interactions in a spatiotemporal manner at distances of 1–10 nm. Competing technologies that claim to assess receptor:ligand interaction (chiefly PD-1/PD-L1 interaction) measure molecule colocalization, which has a working range in the order of micrometers [16]. Furthermore, the correlations calculated above indicate that the checkpoint interactions and ligand expression are not correlated in a statistically significant manner.

Whilst ligand expression does not yield useful information on CTLA-4/CD80 interaction, retrospective studies have identified that an absolute T-lymphocyte count may be associated with patient response to ipilimumab [17]. As there is a documented correlation between T-lymphocyte count and immune checkpoint engagement, we correlated immune checkpoint interactions with CD3+ and CD8+ intratumoral density. It transpired that PD-1/PD-L1 interactions in pre-RFA-treated samples (lung one) significantly negatively correlated to intratumoral CD3+ density. This indicated, with significance, that higher checkpoint interaction was observed in areas with lower CD3+ density. As CD3 is a global T-lymphocyte marker, this could be in part explained by the possibility that not all of the CD3 infiltrate may not be tumor specific. This could also be coupled to the fact that lung metastases are amongst the most infiltrated metastases, and immune infiltrates may be performing other functions, such as response to infection (Supplementary Figure S6) [18]. Nevertheless, the negative correlation of CD8+ with PD1-PDL1 may reflect the state of immune suppression of the RFA-treated patients; that is, a low level of CD8+ and a high interactive state of PD1-PDL1 indicates a high state of immune suppression.

A study has shown that metastases with the least immune-cell infiltration correlate with a prolonged survival, thus highlighting the need to investigate these infiltrates further [19]. Furthermore, more distinct subpopulations of T-lymphocyte infiltration should be defined to assess for a correlation with immune checkpoint engagement or abscopal effect.

### Limitations of This Study

Ideally, the same tumor should have been sampled before and after RFA. As the samples from this cohort were taken (1) from lung one prior to the RFA treatment of lung one and (2) from lung two, post-RFA treatment of lung one and prior to treatment of lung two, intertumoral heterogeneity could not be excluded when comparing results from the pre- and post-RFA biopsies. To sample the same tumor, it would be required to puncture lung two during the first procedure on lung one. Owing to the potential risk of bilateral pneumothorax consecutive to bilateral lung puncture, it was not ethically admissible.

Future studies with a modified biopsy approach could allow iFRET to elucidate further the immune mechanisms that may limit an abscopal effect.

## 5. Conclusions

To summarize, the ability of iFRET to quantify immune checkpoint interactions in patients and determine differences in the dysregulation of different checkpoints may change the paradigm in which immunotherapies are prescribed. Moreover, iFRET has uncovered the need and provided the method for advanced quantitative immune surveyance, as opposed to qualitative immune surveyance, of patients, which will greatly increase patient access to precision medicine. For future studies, to assess the implications of dendritic cells, as well as macrophages, immune surveyance should be performed using imaging mass spectrometry [20].

Other thermal ablative techniques, such as cryoablation, may yield increased antigen release upon tumor treatment; given the wide use of RFA in metastatic colorectal cancer, it is relevant to explore its role in a novel combined modality. As the microsatellite stability of CRC patients can dictate their response to immunotherapies, this could also yield an opportunity to unravel the functional proteomics that underpin immunotherapy resistance in microsatellite stable (MSS) patients [21]. iFRET may hold the potential to elucidate different functional immune checkpoint engagement profiles between MSS patients and microsatellite instable (MSI) patients. There could also be a mechanism whereby microsatellite stability underpins an immune-level resistance to abscopal effects. iFRET precisely determines the functional states of immune check point regulators in RFA-treated patients to render an accurate immunotherapy treatment for this cohort of patients.

## Data Availability

The data presented in this study are available on request from the corresponding author.

## Supplementary Materials

The following supporting information can be downloaded at https://www.mdpi.com/article/10.3390/cancers14235738/s1. Figure S1 Radiofrequency Ablation Procedure Overview; Figure S2. immune-FRET Assay Schematic; Figure S3. Tyramide Signal Amplification; Figure S4. The validation of CTLA-4/CD80 antibodies for use in tissue resulted in the detection of strong interaction states in a mixed lung cancer TMA; Figure S5 CTLA-4/CD80 and PD-1/PD-L1 Interactions do not correlate with intratumoural CD3 or CD8 density Post-RFA (Lung 2); Figure S6. CD3+ and CD8+ intratumoral negative correlation with immune checkpoint regulators.
