# Supplementary material for: Determination of Interactive States of Immune Checkpoint Regulators in Lung Metastases after Radiofrequency Ablation
Source: Cancers (Basel). 2022 Nov 22;14(23):5738. doi: 10.3390/cancers14235738 (PMC9737190; doi:10.3390/cancers14235738)
Supplement: Supplementary file 1 [file cancers-14-05738-s001.zip › cancers-2005155-supplementary.pdf]

cancers MDPI

Supplementary Materials: The following supporting information can be downloaded at: https://www.mdpi.com/article/10.3390/cancers14235738/s1.

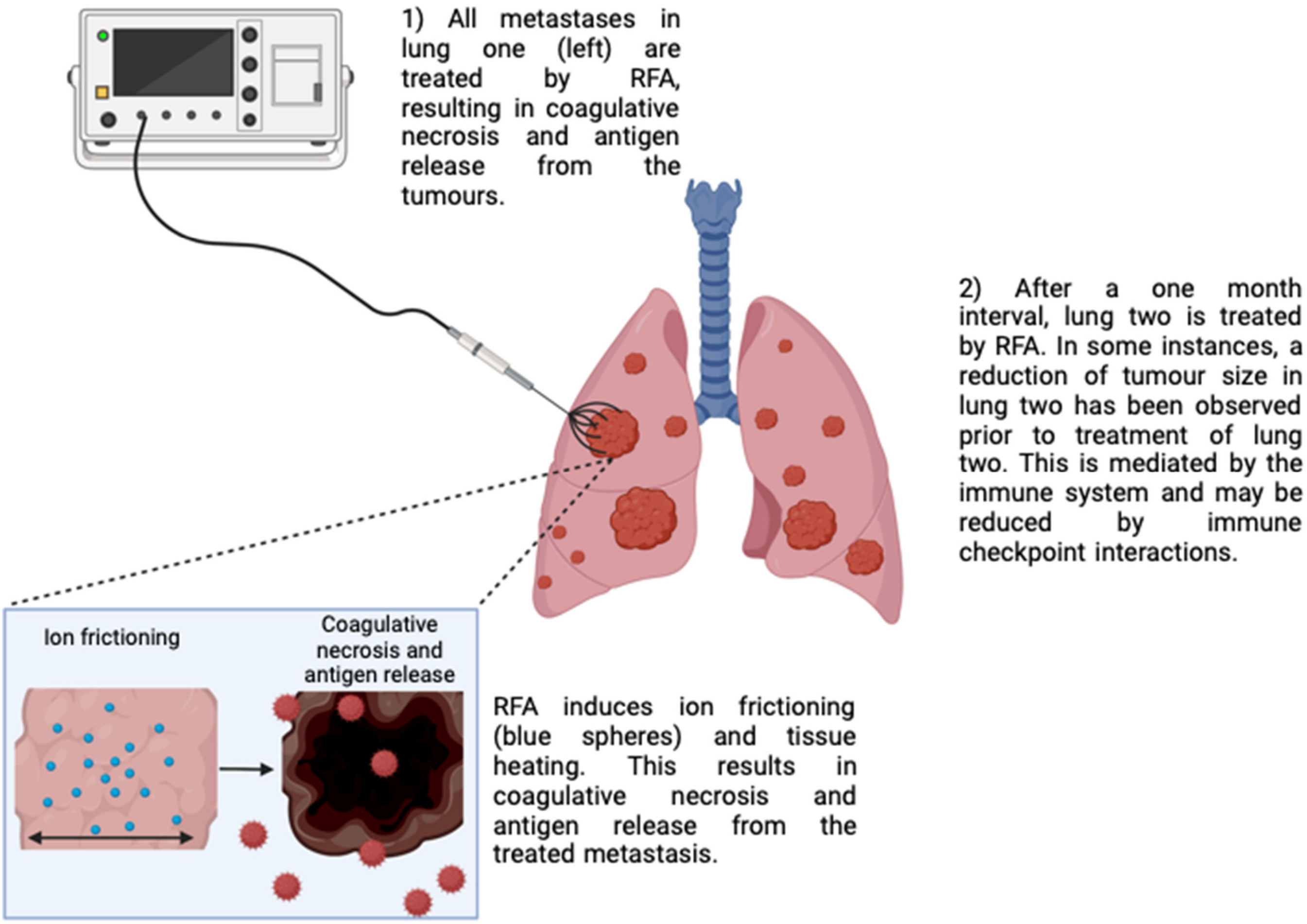

Figure S1. Radiofrequency Ablation Procedure Overview. Radiofrequency ablation (RFA) is able to provide localized treatment to patients with bilateral lung metastases. Firstly (1) all lung metastases in lung number one are treated. The treatment consists of applying a high frequency radiofrequency current to the tumor via a multi-tined electrode. This results in ion fractioning, tissue heating and subsequently coagulative necrosis. This also results in a release of antigen from the tumor. After an interval of one month, the tumors on lung two are treated in the same manner (2). In rare instances, a reduction in the tumors of lung two has been observed after the treatment of lung one. This is known as an abscopal effect and is thought to be, in part, mediated by the adaptive immune system.

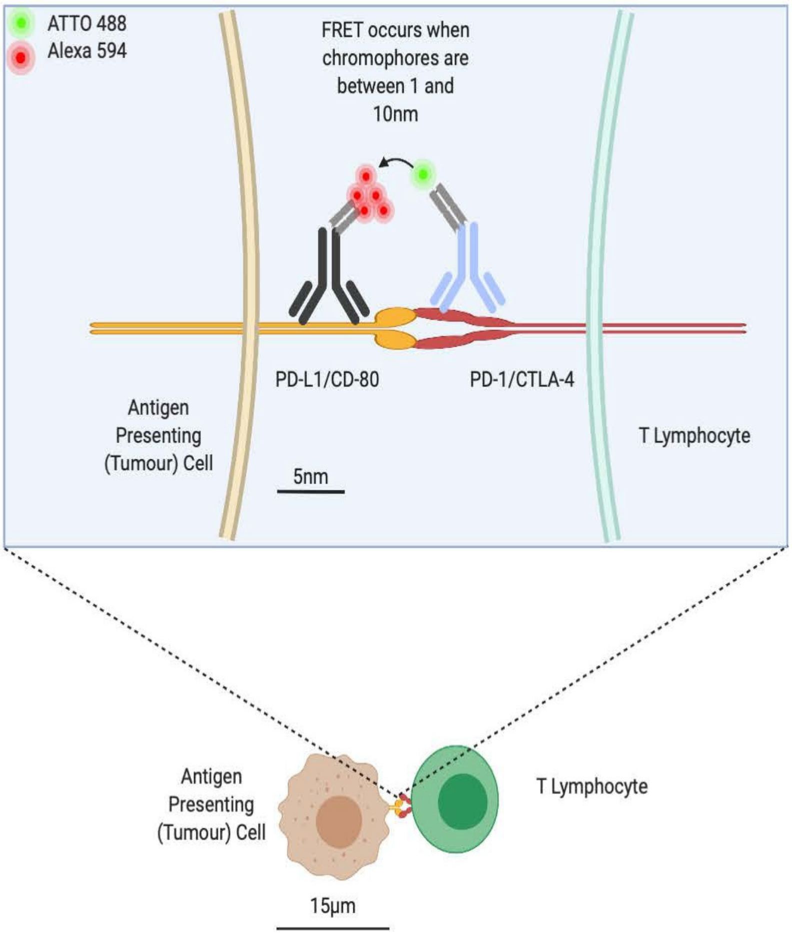

Figure S2. immune-FRET Assay Schematic. iFRET utilizes a two-site assay which determines the interactive states of the immune- checkpoint ligands and receptors. Both the receptor and ligand are labelled with a primary antibody (anti-mouse and anti-rabbit respectively). The anti-mouse primary antibody (detecting CTLA-4 or PD-1) is then labelled with a Fab fragment conjugated to ATTO488, the donor chromophore. The anti-rabbit antibody (detecting CD80 or PD-L1) is then labelled with a (Fab')$_2$ fragment conjugated to horseradish peroxidase (HRP). Using tyramide signal amplification, the HRP labels the sample with Alexa594 which acts as the acceptor chromophore. FRET can only occur at distances of 1-10nm; the use of secondary Fab fragments allows the critical FRET distance of 10nm to be maintained. In the schematic shown here, the ligand and receptor are interacting.

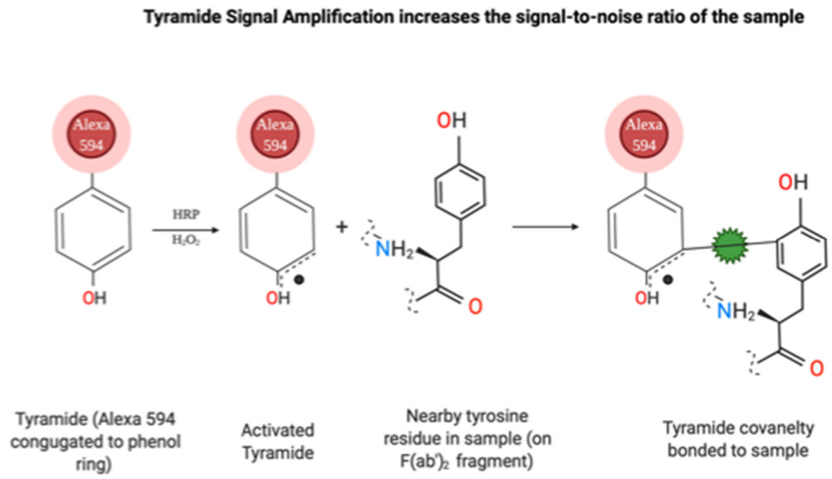

Figure S3. Tyramide Signal Amplification. To observe a good signal to noise ratio, tyramide signal amplification is performed on all samples. The donor acceptor site (PD-L1 or CD80) is labelled with a primary antibody which is labelled with a secondary F(ab′)2 fragment conjugated to horseradish peroxidase (HRP). When tyramide (a phenol ring conjugated to Alexa594 at the para position) is added to the sample in the presence of H_2O_2, a free radical is generated. The tyramide then covalently bonds to nearby tyrosine residues in the sample (on the F(ab′)2 fragment). An incubation time of 10 minutes is used to prevent non-specific binding of the chromophore to the sample.

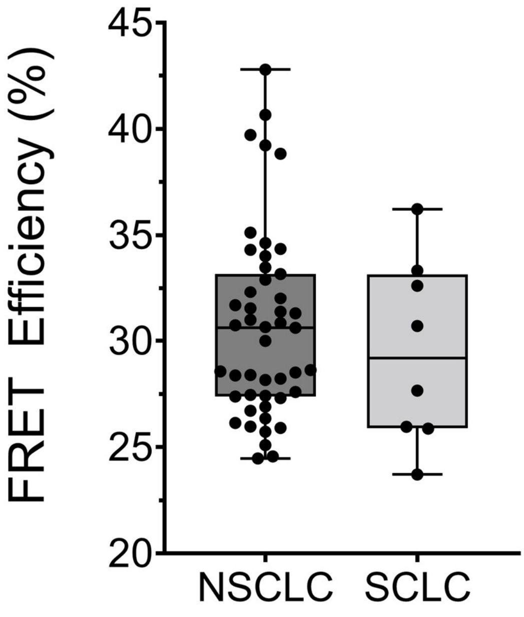

Figure S4. The validation of CTLA-4/CD80 antibodies for use in tissue resulted in the detection of strong interaction states in a mixed lung cancer TMA. As the CTLA-4/CD80 antibody dilutions, utilized in Sánchez-Magraner et al., 2020, had not been validated in tissue before, a commercial TMA containing a mix of lung carcinoma samples was used to validate antibody dilutions. The antibody dilutions used were 1:100 for both antibodies. The box and whisker plot has categorized the carcinomas as non-small cell lung carcinoma or small-cell lung carcinoma. Strong positive FRET efficiencies were observed across both groups, indicating a strong CTLA-4/CD80 binding in the analyzed samples. The median FRET efficiencies observed were 30.62% and 29.19% for NSCLC and

SCLC groups, respectively. A population of high interaction states are observed in the upper quartile of both groups.

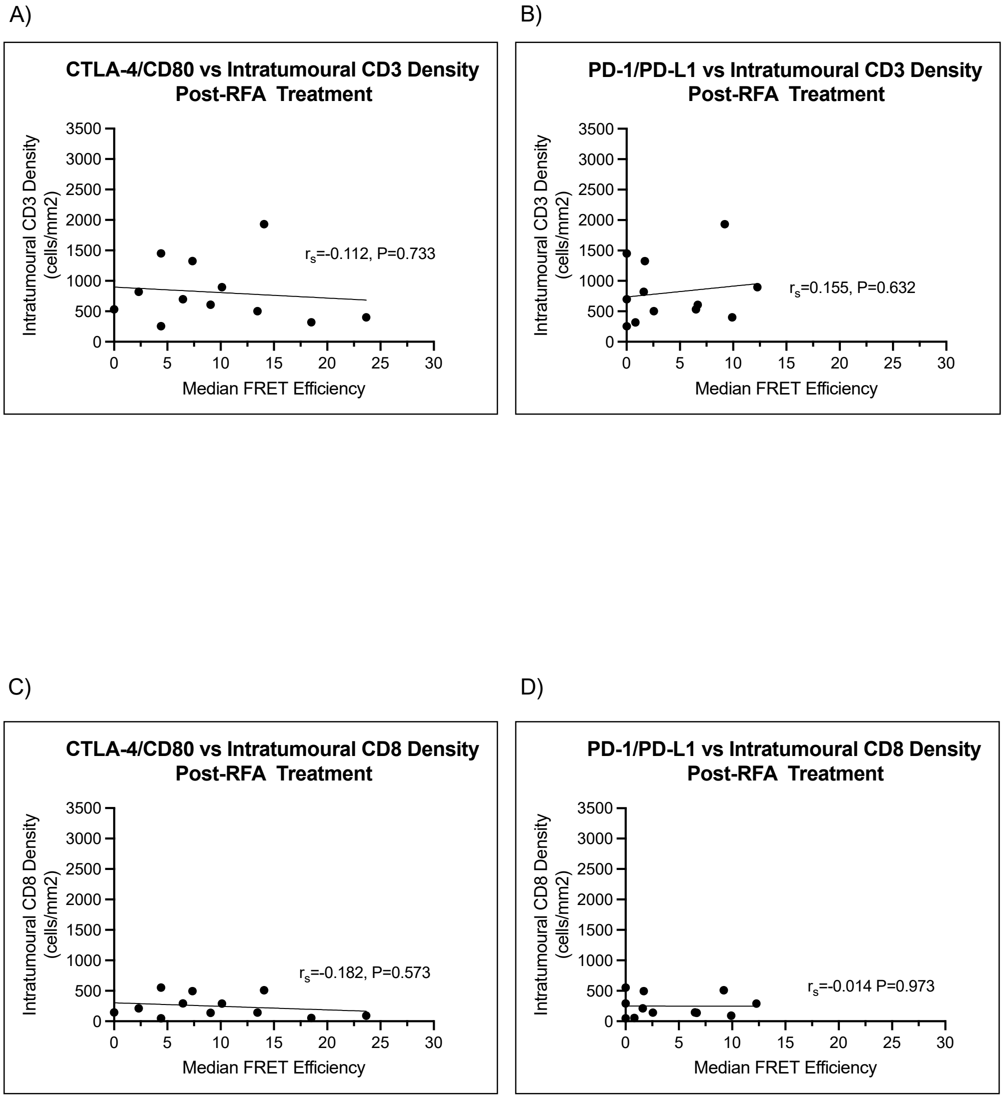

Figure S5. CTLA-4/CD80 and PD-1/PD-L1 Interactions Do Not Correlate with Intratumoural CD3 or CD8 Density Post-RFA (Lung 2). A) Median CTLA-4/CD80 interactions from lung 2 (post-RFA) failed to correlate with intratumoral CD3 density (r_s=-0.112, P=0.733). **B)** A lack of correlation was also seen between median PD-1/PD-L1 interaction and intratumoral CD3 density in metastases from lung 2 (r_s=0.155, P=0.632). Both median CTLA-4/CD80 interaction states (**C**) and median PD-1/PD-L1 interaction states (**D**) failed to correlate with intratumoral CD8 density in metastases from lung 2 (r_s=-0.182, P=0.573 and r_s=-0.014, P=0.973, respectively).

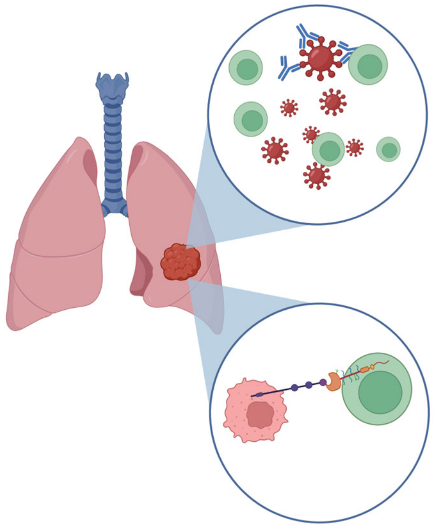

Figure S6. CD3+ and CD8+ intratumoral negative correlation with immune checkpoint regulators. Whilst CD3+ cells infiltrate the tumour, these cells may be naïve to tumour specific antigens. In this case, lymphocyte infiltration will not result in a lymphocyte-mediated attack of the tumour. Additionally, CD3+ infiltration may report on lymphocytes performing other functions within the lung, such as the clearance of infection. CD8+ and interactive states of PD-1/PD-L1 can be used to assess the state of immune suppression in RFA treated patients.
